# Toxoplasmosis-Associated Difference in Intelligence and Personality in Men Depends on Their Rhesus Blood Group but Not ABO Blood Group

**DOI:** 10.1371/journal.pone.0061272

**Published:** 2013-04-10

**Authors:** Jaroslav Flegr, Marek Preiss, Jiří Klose

**Affiliations:** 1 Department of Biology, Faculty of Science, Charles University in Prague, Prague, Czech Republic; 2 Department of Biochemistry & Brain Pathophysiology, Prague Psychiatric Center, Prague, Czech Republic; 3 Central Medical Psychology Department, Military University Hospital Prague, Prague, Czech Republic; Karolinska Institutet, Sweden

## Abstract

**Background:**

The parasite *Toxoplasma gondii* influences the behaviour of infected animals and probably also personality of infected humans. Subjects with a Rhesus-positive blood group are protected against certain behavioural effects associated with *Toxoplasma* infection, including the deterioration of reaction times and personality factor shift.

**Methodology/Principal Findings:**

Here, we searched for differences in the toxoplasmosis-associated effects between RhD-positive and RhD-negative subjects by testing 502 soldiers with two personality tests and two intelligence tests. The infected subjects expressed lower levels of all potentially pathognomic factors measured with the N-70 questionnaire and in neurasthenia measured with NEO-PI-R. The RhD-positive, *Toxoplasma*-infected subjects expressed lower while RhD-negative, *Toxoplasma*-infected subjects expressed higher intelligence than their *Toxoplasma*-free peers. The observed *Toxoplasma*-associated differences were always larger in RhD-negative than in RhD-positive subjects.

**Conclusions:**

RhD phenotype plays an important role in the strength and direction of association between latent toxoplasmosis and not only psychomotor performance, but also personality and intelligence.

## Introduction

The trophically transmitted parasites often modify the behavior of their intermediate host to increase its susceptibility to predation [1], [2]. By this they increase the probability of their transmission from intermediate to definitive host. A popular model for studying such manipulation activity of parasites in a mammal host is *Toxoplasma gondii*, for review see [3], [4]. In its life cycle, *Toxoplasma* needs to be transmitted from the intermediate host, e.g. an infected rodent, to the definitive host, i.e. any representative of the Felinidae family, including the domestic cat. It is known that infected rodents are hyperactive in the open field [5], [6], exhibit increased voluntary wheel running [7], [8] and longer exploration times in the hole board test [9], are deficient in motor performance and coordination [8], [10], and have longer reaction times [11], impaired working memory [12], and impaired ability to recognise novel stimuli [8], [13]. The most specific and also the most spectacular toxoplasmosis-associated change reported in rodents is the so-called Fatal attraction phenomenon, i.e. the conversion of the rats' and mice's innate fear of cat odour into attraction to cat odour (but not to the odour of other predators). This phenomenon was observed in several laboratories [12], [14]–[16] and was dependent on activation of the brain regions that respond to sexual stimuli in normal mice by the odour of a particular predator, the cat in infected rodents [16]. Current results suggest that changed concentrations of testosterone [17] and dopamine probably play an important role in the differences in the personality and behavior between *Toxoplasma*-infected and *Toxoplasma*-free subjects. It was found that the *Toxoplasma gondii* genome contains two genes for enzymes (tyrosine hydroxylases) implicated in the synthesis of dopamine [18] and increased concentration of this neurotransmitter was observed in the infected rodent brain areas [19].

Any warm-blooded animal, including humans, can be infected with *Toxoplasma* and the prevalence of this infection in different countries varies between 5 and 80% depending on climate, hygienic standards and kitchen habits [20]. After a short phase of acute toxoplasmosis, the infection proceeds to its latent stage when tissue cysts with bradyzoites are formed and these survive for the rest of the host's life mainly in neural and muscular tissues. In immunocompetent subjects, the latent phase of infection was considered asymptomatic and harmless from the clinical point of view, however, results of many recent studies suggested that this form of the infection could have many serious clinical implications [21]–[24]. However, practically all studies performed in the past 20 years have demonstrated behavioural changes including the Fatal attraction phenomenon [25], observed earlier in laboratory animals, also in humans, for recent reviews, see [3], [26].

It is well known that the gene pool of the local human population is strongly influenced by the selection pressure of parasites. Recent studies have shown that the association between latent toxoplasmosis and human reaction times, personality and physiology depend on RhD phenotype of the infected subject [27]–[31]. It has even been suggested that the spreading of the deletion responsible for RhD negativity in the Caucasian population can be caused by increased psychomotor performance of RhD-negative, *Toxoplasma*-free subjects in Europe where the cats and therefore also toxoplasmosis were rare before the advent of the domestic cat [27]. The association between toxoplasmosis and the personality of RhD-negative and RhD-positive subjects was studied using Cattell's 16PF and Cloninger's TCI questionnaires [29]. In the present study, we searched for the difference between RhD-positive and RhD-negative subjects using the NEO-PI-R questionnaire that is based on the modern Big Five model of personality. Moreover, we searched for similar RhD phenotype- and toxoplasmosis-associated differences in verbal and nonverbal intelligence and also in pathognomic traits measured with the N-70 questionnaire.

## Materials and Methods

### Ethics Statement

All participants provided their written informed consent. The recruitment of study subjects and data handling were performed in compliance with the Czech legislation in force and were approved by the Institutional Review Board of the Faculty of Science, Charles University.

### Sample and Participant Selection

All psychological testing was performed at the Military University Hospital Prague. The study population consisted of 502 male soldiers of Czech nationality (age: 18–52, mean 27.25, S.D. 6.71, median 25.94) who attended the Military University Hospital Prague to take entrance psychological examinations for military missions in 2005 and consented to participate in the research project. The subjects were examined with standardized panel of psychological and performance tests, essayed for RhD and ABO phenotype during the health examination and also provided 5 ml of blood for a serology test. In the informed consent form, the general aim of the project (a study of the influence of environmental factors on human psychology and performance) and the need for obtaining their consent to using the results of their psychological and clinical examinations were explained. The consent rate was about 65%.

### N-70 questionnaire

The N-70 is a questionnaire constructed for the assessment of seven areas of clusters - anxiety, depression, phobia, hysteria, hypochondria, psychosomatic symptoms and psychastenia [32]. The purpose of this method is to detect individuals who may be too sensitive for military operations [33]. Subjects are asked to answer 70 questions using a 3-point agreement scale. Scores in each cluster range from 0–30. The total N-70 score is the number of non-negative answers for all 70 questions.

### NEO-PI-R questionnaire

The electronic version of NEO-PI-R (Costa & Mccrae, 1992) translated to Czech and validated by Hřebíčková (2001) [34] was used.

### Wiener Matrizen-Test of intelligence

The Wiener Matrizen-Test (WMT) [35], a nonverbal intelligence test, is an adapted version of the Raven progressive matrices which conforms to the Rasch model [36]. The WMT assesses general intelligence by measuring reasoning ability. The test requires the completion of 24 matrices with increasing task difficulty and was administered without an explicit time limit. The intention and conceptualization of the WMT are largely based on Raven's Matrices [37]–[39]. The correlation between the WMT and Standard Progressive Matrices is about r = 0.92 [35]. Construction and item selection, however, follow the standards of Rasch scaling. For these reasons, and due to the fact that the WMT showed comparable validity characteristics but had a considerably higher administration economy, we prefer the WMT to the Raven matrices in clinical practice. The split-half reliability of the WMT is 0.83 [35]. The 1993 Czech adopted version [40], distributed by Psychodiagnostika (Brno), was used in the present study. Both the raw score and the IQ adjusted for age of the participant were compared in statistical tests.

### OTIS test of intelligence

The OTIS test is a test of verbal intelligence which was derived from the original test [41]. Seven types of items were taken from the original test:

- term or object definition by choosing the most suitable characteristics
- term or object definition by choosing the most suitable description
- the choice of an object based on common attributes
- the choice of the opposite
- the identifying of “foreign” (unrelated) terms
- logical or ethical solution of the situations
- the interpretation of the adage

The test contains 32 items (0–32). The maximum score is therefore 32 points. Both the raw score and the IQ (adjusted for the educational level, see [32]) were compared in statistical tests.

### Immunological tests for toxoplasmosis

All serological tests were carried out in the National Reference Diagnostic Laboratory for Toxoplasmosis, National Institute of Public Health, Prague. Specific IgG and IgM antibody titres were determined by ELISA (IgG: SEVAC, Prague, IgM: TestLine, Brno), optimized for early detection of acute toxoplasmosis (Pokorný *et al.*, 1989) and by complement fixation tests (CFT) (SEVAC, Prague) which are more sensitive and therefore more suitable for the detection of old *T. gondii* infection (Warren & Sabin, 1942). The titre of anti-*Toxoplasma* antibodies in sera was measured in dilutions between 1∶8 and 1∶1024. The subjects with negative results of IgM ELISA (positivity index<0.9) and both CFT titres higher than 1∶8 and IgG ELISA >250 optical units, i.e. approximately 10 IU/ml, were considered latent toxoplasmosis positive. The individuals with ambiguous diagnosis, e.g. different result of CFT and ELISA, were excluded from the study.

### Statistical analysis

The Statistica 8.0 was used for descriptive statistics, General Linear Model tests and computing *t au*s by standard Kendall correlation tests. Partial Kendall correlation test suggested by Siegel and Castellan [42] based on *tau*s computed with standard Kendall correlations was used for nonparametric analyses [17]; the Excel sheet for this analysis is available at http://web.natur.cuni.cz/flegr/programy.php.

## Results

**Figure 1:**
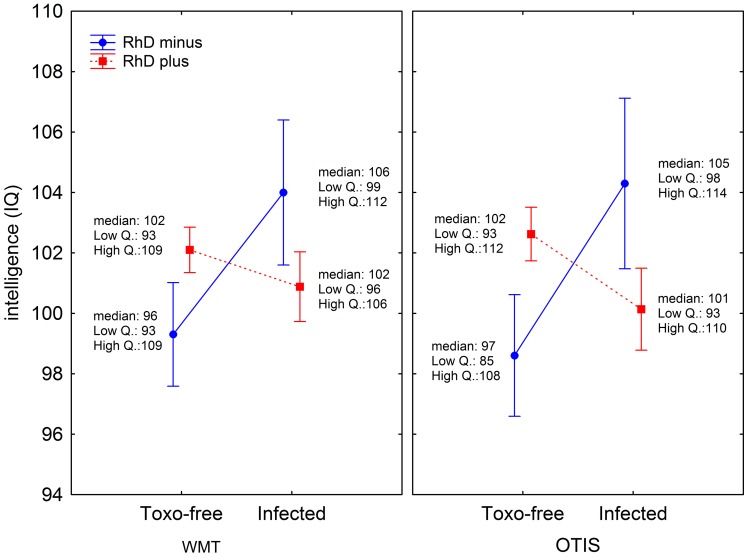
Differences in nonverbal (WMT) and verbal (OTIS) intelligence between *Toxoplasma*-infected and *Toxoplasma*-free RhD-positive and RhD-negative subjects. The graph shows arithmetic means, standard errors (whiskers), medians, and 25% and 75% quartiles. The presented values differ from raw data listed in Tables 1 and 2 because the intelligence has been controlled for the age of men, that is, the intelligence has been computed for covariate (age) as its mean.

We obtained scores for the N-70, NEO-PI-R, WMT and Otis tests from 502 subjects tested for RhD and latent toxoplasmosis. One hundred and fifty-four (154, i.e. 31.4%) of 491 subjects with unambiguous results of the test for toxoplasmosis were *Toxoplasma* infected and 87 (17.3%) of 502 subjects were RhD negative. No association between toxoplasmosis and RhD phenotype was observed (Chi^2^ = 0.14, p = 0.707). Descriptive statistics for the population under study are shown in Tables 1 and 2. For the analysis of correlation of toxoplasmosis and RhD phenotype with the personality profile of soldiers (ordinal variables), we used a robust nonparametric test. To control for the effect of age, partial Kendal correlation tests were performed with age as a covariate and to control for the effect of RhD phenotype, RhD-positive and RhD-negative subjects were tested separately. Table 1 shows that *Toxoplasma*-infected subjects scored lower in the total N-70 score and also in anxiety, depression, phobia, hysteria, and vegetative lability and in the BigFive trait neuroticism. The differences were much stronger in RhD-negative than RhD-positive subjects. No relation between latent toxoplasmosis and nonverbal (WMT) or verbal (Otis) intelligence was observed in RhD nonsorted population. However, separate analyses performed for RhD-positive and RhD-negative populations showed negative association between intelligence and toxoplasmosis in RhD-positive subjects and positive association between intelligence and toxoplasmosis in RhD-negative subjects, see Fig. 1. Again, the correlation of intelligence with toxoplasmosis (estimated with partial *tau*) was much stronger for RhD-negative subjects.

**Table 1:** Descriptive statistics and results of testing differences in personality traits and intelligence between *Toxoplasma*-infected and *Toxoplasma*-free RhD-negative and RhD- positive male soldiers.

			All						Rh+						Rh−			
	N	mean			N	mean			N	mean								
	Toxo−	Toxo+	Toxo−	Toxo+	Tau	p	Toxo−	Toxo+	Toxo−	Toxo+	Tau	p	Toxo−	Toxo+	Toxo−	Toxo+	Tau	p
Age	337	154	26.70	27.83			280	125	26.77	28.26			57	28	26.35	26.26		
Total N-70	335	152	18.97	16.57	−0.09	**0.002**	278	123	18.97	17.21	−0.07	**0.032**	57	28	18.96	13.50	−0.23	**0.002**
Anxiety	335	152	4.31	3.68	−0.10	**0.001**	278	123	4.28	3.74	−0.09	**0.009**	57	28	4.47	3.39	−0.19	**0.009**
Depression	335	152	2.18	1.80	−0.07	**0.018**	278	123	2.16	1.86	−0.05	0.102	57	28	2.26	1.50	−0.18	**0.016**
Phobia	335	152	2.87	2.50	−0.07	**0.024**	278	123	2.86	2.57	−0.06	0.096	57	28	2.95	2.14	−0.13	0.069
Hysteria	335	152	2.87	2.34	−0.11	**0.000**	278	123	2.91	2.42	−0.10	**0.002**	57	28	2.68	2.00	−0.12	0.095
Hypochondria	335	152	2.51	2.24	−0.06	0.062	278	123	2.54	2.36	−0.03	0.295	57	28	2.40	1.71	−0.16	**0.031**
Vegetative lability	335	152	3.15	2.71	−0.08	**0.013**	278	123	3.18	2.82	−0.06	0.089	57	28	3.00	2.18	−0.16	**0.033**
Psychasteny	335	152	2.13	1.91	−0.05	0.074	278	123	2.14	2.10	−0.02	0.566	57	28	2.11	1.00	−0.24	**0.001**
Neuroticism	314	143	69.06	65.15	−0.07	**0.025**	259	117	68.86	66.49	−0.04	0.253	55	25	69.98	59.32	−0.22	**0.005**
Extroversion	314	143	116.84	116.70	0.00	0.887	259	117	116.79	116.83	0.01	0.678	55	25	117.11	116.80	−0.00	0.957
Openness	314	143	101.81	102.40	−0.00	0.943	259	117	102.89	101.41	−0.03	0.393	55	25	100.47	106.88	0.130	0.089
Agreeableness	318	144	122.42	123.45	0.03	0.265	259	117	121.80	123.28	0.05	0.174	55	25	125.35	123.68	−0.03	0.689
Conscientiousness	316	144	128.81	130.07	0.03	0.265	259	117	128.73	130.12	0.04	0.272	55	25	129.18	130.00	0.03	0.745
Row WMT	314	143	23.19	23.04	−0.01	0.695	278	123	23.38	22.72	−0.07	**0.044**	57	28	22.28	24.46	0.24	**0.001**
IQ WMT	312	142	101.49	101.09	−0.01	0.712	278	123	101.95	100.37	−0.06	0.083	57	28	99.25	104.54	0.21	**0.004**
Row Otis	311	141	14.53	14.12	−0.03	0.368	273	117	14.75	13.86	−0.07	**0.027**	53	27	13.38	15.41	0.21	**0.007**
IQ Otis	309	140	101.97	100.78	−0.02	0.595	273	117	102.62	100.14	−0.06	0.099	53	27	98.60	104.30	0.18	**0.017**

*Tau* shows effect size and sign, p shows statistical significance measured with partial Kendall tests. Significant results (p<0.05, two-sided test) are printed in bold. *Toxoplasma*-free and *Toxoplasma*-infected subjects are coded with 0 and 1, respectively. Therefore, negative Tau means lower test score in *Toxoplasma* infected subjects. Formal correction for multiple (51) tests was not performed. Theoretically, 2–3 of 51 tests presented in this table should provide false positive results.

**Table 2:** Descriptive statistics and results of testing differences in personality traits and intelligence between RhD-negative and RhD-positive *Toxoplasma*-infected and *Toxoplasma*-free male soldiers.

			All						Toxo−						Toxo+			
	N	mean			N	mean			N	mean								
	RhD−	RhD+	RhD−	RhD+	Tau	p	RhD−	RhD+	RhD−	RhD+	Tau	p	RhD−	RhD+	RhD−	RhD+	Tau	p
Age	87	415	26.36	27.26			57	280	26.35	26.77			28	125	26.26	28.26		
Total N-70	87	411	16.97	18.36	0.05	0.108	57	278	18.96	18.97	0.00	0.980	28	123	13.50	17.21	0.12	**0.027**
Anxiety	87	411	4.08	4.11	0.00	0.890	57	278	4.47	4.28	−0.03	0.440	28	123	3.39	3.74	0.06	0.303
Depression	87	411	1.97	2.04	0.00	0.869	57	278	2.26	2.16	−0.04	0.291	28	123	1.50	1.86	0.05	0.401
Phobia	87	411	2.66	2.75	0.03	0.377	57	278	2.95	2.86	0.00	0.979	28	123	2.14	2.57	0.07	0.189
Hysteria	87	411	2.44	2.74	0.06	0.057	57	278	2.68	2.91	0.05	0.194	28	123	2.00	2.42	0.07	0.204
Hypochondria	87	411	2.18	2.46	0.05	0.108	57	278	2.40	2.54	0.02	0.565	28	123	1.71	2.36	0.12	**0.029**
Vegetative lability	87	411	2.68	3.08	0.06	**0.050**	57	278	3.00	3.18	0.02	0.666	28	123	2.18	2.82	0.13	**0.021**
Psychasteny	87	411	1.70	2.13	0.09	**0.003**	57	278	2.11	2.14	0.02	0.655	28	123	1.00	2.10	0.21	**<0.001**
Neuroticism	82	386	57.90	59.16	0.03	0.314	55	259	60.75	59.72	−0.02	0.639	25	117	51.92	58.01	0.13	**0.018**
Extroversion	82	386	95.21	95.52	0.01	0.827	55	259	95.20	95.52	−0.01	0.843	25	117	94.84	95.44	0.05	0.344
Openness	82	386	82.70	82.22	−0.01	0.814	55	259	81.24	82.51	0.02	0.600	25	117	86.28	81.80	−0.06	0.270
Agreeableness	82	386	104.63	102.00	−0.06	**0.045**	55	259	105.45	101.64	−0.08	**0.027**	25	117	103.08	103.03	−0.01	0.838
Conscientiousness	82	386	109.18	108.84	−0.01	0.763	55	259	108.75	108.31	−0.01	0.795	25	117	109.72	109.65	−0.01	0.905
Row WMT	87	411	22.94	23.20	0.03	0.362	57	278	22.28	23.38	0.09	**0.010**	28	123	24.46	22.72	−0.15	**0.005**
IQ WMT	87	411	100.79	101.55	0.03	0.347	57	278	99.25	101.95	0.08	**0.027**	28	123	104.54	100.37	−0.13	**0.016**
Row Otis	82	399	13.93	14.45	0.05	0.094	53	273	13.38	14.75	0.12	**0.002**	27	117	15.41	13.86	−0.12	**0.030**
IQ Otis	82	399	99.99	101.76	0.05	0.137	53	273	98.60	102.62	0.10	**0.007**	27	117	104.30	100.14	−0.11	**0.047**

*Tau* shows effect size and sign, p shows statistical significance measured with partial Kendall. RhD-negative and RhD-positive subjects are coded with 0 and 1, respectively. Therefore, negative Tau means lower test score in RhD-positive subjects. Formal correction for multiple (51) tests was not performed. Theoretically, 2–3 of 51 tests presented in this table should provide false positive results.

The same analyses (partial Kendall correlations with age as a covariate) was performed for the independent binary variable RhD phenotype, in the whole population and separately in the *Toxoplasma*-infected and *Toxoplasma*-free subjects, see Table 2. Significant association of RhD phenotype with the total N-70 score, hypochondria, vegetative lability, psychasteny, and the NEO-PI-R neuroticism were observed only in *Toxoplasma*-infected subjects. However, the association of RhD phenotype with nonverbal and verbal intelligence was detected also in *Toxoplasma*-free subjects, suggesting that not only the protective effect of RhD positivity against consequences of toxoplasmosis but also the main effect of RhD phenotype (or its protective effect against some unknown third factor) probably played a role in the observed associations between RhD phenotype and various personality traits.

The partial Kendall correlation test can control for one confounding variable only. To study the effect of interactions and several potential confounding variables we performed General Linear Model analyses with independent variables age, toxoplasmosis, RhD phenotype, ABO phenotype and RhD phenotype-toxoplasmosis and ABO phenotype-toxoplasmosis interactions. The analyses showed significant effect of RhD phenotype-toxoplasmosis interaction on psychasteny and IQ and no significant effects of ABO phenotype or ABO phenotype interaction (Tab. 3).

**Table 3:** Results of testing the effects of age, toxoplasmosis, RhD phenotype, ABO phenotype, and RhD-toxoplasmosis and ABO-toxoplasmosis interaction on personality traits and intelligence.

	age	ABO	RhD	Toxo	ABO-Toxo	RhD-Toxo
Total N-70	0.819	0.677	0.076	**0.034**	0.807	0.149
Anxiety	0.999	0.637	0.541	**0.032**	0.578	0.395
Depression	0.467	0.476	0.513	0.119	0.798	0.392
Phobia	0.410	0.674	0.353	0.121	0.907	0.289
Hysteria	0.674	0.992	0.237	0.089	0.677	0.560
Hypochondria	0.522	0.168	**0.016**	0.174	0.378	0.280
Vegetative lability	0.647	0.765	0.188	0.168	0.685	0.470
Psychasteny	0.287	0.552	0.936	0.148	0.163	**0.019**
Neuroticism	0.443	0.846	0.200	0.052	0.791	0.164
Extroversion	0.825	0.793	0.964	0.671	0.336	0.999
Openness	0.618	0.483	0.471	0.549	0.562	0.144
Agreeableness	0.447	0.844	0.357	0.493	0.848	0.264
Conscientiousness	0.863	0.351	0.903	0.746	0.562	0.760
Row WMT	0.308	0.243	0.651	0.203	0.794	**0.010**
IQ WMT	0.422	0.237	0.806	0.317	0.943	**0.037**
Row Otis	0.167	0.611	0.783	0.081	0.132	**0.003**
IQ Otis	0.287	0.551	0.936	0.148	0.163	**0.019**

The table shows p-values of particular GLM tests. Significant results (p<0.05, two-sided test) are printed in bold. Formal correction for multiple tests was not performed. Theoretically, about one false positive result should be present in each column.

## Discussion

Soldiers with and without latent *Toxoplasma* infection differ in several personality traits. Generally, the infected subjects expressed lower levels of potentially pathognomic factors measured with the N-70 questionnaire and of neuroticism tested with the NEO-PI-R (Big Five model). The RhD-positive, *Toxoplasma*-infected subjects express lower while RhD-negative, *Toxoplasma*-infected subjects express higher verbal and nonverbal intelligence than their *Toxoplasma*-free peers. The observed *Toxoplasma*-associated differences in personality traits, including intelligence were always larger in RhD-negative than in RhD-positive subjects.

The GLM analysis showed that the effect of RhD-toxoplasmosis interaction on intelligence is highly significant. This analysis also showed a significant effect RhD-toxoplasmosis interaction on psychasteny. It must be reminded, however, that this effect is non-significant after the correction for multiple statistical tests. The GLM also showed absence of main effects of RhD phenotype and toxoplasmosis (after correction for multiple tests), which contrasted with results of partial Kendall correlation tests. The lower power of parametric tests for ordinal data with asymmetric distribution as well as the presence of several other independent variables and their interactions in more complex GLM models could be responsible for this difference between results of parametric and nonparametric tests. GLM analysis models also showed absence of effect of ABO phenotype and its interaction on personality and intelligence. Absence of any effect of ABO phenotype contrasted with existence of numerous effects of RhD phenotype – see the Table 3, confirming the special role of RhD proteins in human physiology.

Association between *Toxoplasma* infection and human personality factors were studied thoroughly in the past 20 years. About 10 published studies have demonstrated associations of toxoplasmosis with human personality traits mostly using Cattell's 16PF and Cloninger's TCI questionnaires; for review, see [26], [43], [44]. Only one study, showing positive association of toxoplasmosis with extroversion and its negative association with conscientiousness, used the NEO-PI-R questionnaire [45]. A correlation study has also shown that the difference in the prevalence of latent toxoplasmosis between the general populations of particular countries can explain a significant portion of the variance in aggregate neuroticism among populations [46].

Surprisingly, the results obtained in the present study performed on military personnel differed from those observed earlier on university students. For example, *Toxoplasma*-infected and *Toxoplasma*-free soldiers expressed no differences in extroversion or conscientiousness and *Toxoplasma*-infected and *Toxoplasma*-free students expressed no difference in neuroticism. Moreover, the results of the correlation study comparing the prevalence of latent toxoplasmosis with aggregate neuroticism in the general populations of particular countries suggest that *Toxoplasma*-infected subjects have higher rather than lower neuroticism [46]. It was also suspicious that infected soldiers expressed lower and not higher levels of psychopathognomic traits measured with the N-70 questionnaire. Our present hypothesis is that *Toxoplasma*-infected soldiers express stronger tendency to mask any negative property when responding to questions in questionnaires. Several studies have shown a lower superego strength (Cattell's factor G) and higher suspiciousness (Cattell's factor L) in *Toxoplasma*-infected men. The testing of soldiers in the current study was a part of their entrance examination for a voluntary (and well-paid) participation in an international military mission and (in contrast with university students or blood donors tested in the previous anonymous studies) the subjects were objectively motivated to mask their negative (e.g. the pathognomic) and to accentuate their positive properties. It is urgently needed to confirm our results in an anonymous research study where the motivation for intentional distortion of data is lower.

Existence of the interaction between toxoplasmosis, RhD phenotype and human behaviour has been confirmed in four studies. Two of them have shown resistance of RhD-positive subjects, especially the RhD-positive heterozygotes, to impairment of reaction times after *Toxoplasma* infection [27], [28] and one prospective study performed on 3900 military drivers has found an increased risk of traffic accidents in *Toxoplasma*-infected, RhD-negative subjects [30]. The fourth study has reported opposite relation of toxoplasmosis with Cattell's ego strength, praxernia, and ergic tension and Cloninger's cooperativeness in RhD-positive and RhD-negative blood donors [29]. The latter study also indicates that RhD phenotype might play an important role not only in the toxoplasmosis-associated differences but also in the age-associated differences in specific personality traits.. Another recent study shows that RhD phenotype could also play a role in correlations of age and smoking with psychomotor performance, intelligence and health of draftees [47]. The results of the current study are in an agreement with the already published data. The correlations of toxoplasmosis with personality of soldiers (reflected by the absolute values of Kendall *tau* shown in Tables 1 and 2) were always much stronger in RhD-negative than RhD-positive subjects, see Table 1. Moreover, the higher verbal and nonverbal intelligence of RhD-positive *Toxoplasma*-free subjects than Rh-negative *Toxoplasma*-free soldiers suggests that RhD positivity could protect not only against detrimental effects of latent toxoplasmosis but also against other (still unknown) factors. At the present time, we have no explanation for the opposite relation between RhD phenotype and intelligence in *Toxoplasma*-infected and *Toxoplasma*-free subjects. We cannot exclude a possibility that some unknown gene that is in linkage disequilibrium with RHD gene, rather than RHD gene itself, is responsible for the observed phenomena. We cannot even exclude a possibility that the observed phenomena are caused by some unknown confounding variables that co-vary with RhD phenotype and also other observed variables, namely risk of *Toxoplasma* infection and human personality and intelligence. However, the present data could explain the controversial results concerning the existence (and direction) of the correlation between latent toxoplasmosis on intelligence [48], [49].

The mechanism responsible for physiological and behavioural effects of RhD phenotype is unknown. The RhD molecule is part of a molecular complex (RhAG) on the membrane of red cells [50], [51]. Structural data suggest that the complex is a membrane NH_3_ or possibly CO_2_ pump with unknown function [52]–[54]. In RhD-negative subjects, the gene RHD is absent in chromosomes of both maternal and paternal origin due to a large deletion and therefore also the RhD molecule is missing and is probably substituted with another related molecule in the complex [55]. RhD-containing and RhD-free complexes may differ in the specificity, activity and most probable also response to regulation signals. The membrane pump could directly or indirectly influence the partial tension of oxygen and water balance in various tissues, including the brain tissue [56]–[58].

### Limitations of the present study

The major limitation of the present study was that the study subjects were objectively motivated to accent positive and to hide negative traits of their personality as their results were to be used as a part of the entrance examination for the participation in a military (peacekeeping) mission. The resulting bias probably cannot influence the result of the intelligence tests; however, it makes it difficult to interpret psychological meanings of the observed relations of toxoplasmosis and RhD phenotype with the personality profile. Many subjects were probably aware about their RhD phenotype; however, nobody was aware either about the hypothesis under study or about their toxoplasmosis status and therefore no systematic bias in the obtained data could be expected.

The second important limitation of the study was the fact that only RhD phenotype and not RhD genotype of the subjects was tested. Results of a previous study suggested that in contrast to RhD-positive heterozygotes, the RhD-positive homozygotes were only transiently protected against some negative effects of toxoplasmosis (namely against prolongation of reaction times) [27]. It is very easy (and cheap) to determine RhD phenotype using the standard agglutination technique. However, a much more sophisticated (and expensive) technique must be used for the determination of RhD genotype. It is also highly probable that a much lower fraction of the soldiers would consent to be involved in a study that would include also DNA analysis. Due to these technical limitations, we compared RhD-negative homozygotes with a mixed population of RhD-positive homozygotes and heterozygotes in all our statistical tests. It is therefore possible that we underestimated the strength of real effects. Only male soldiers were included into the present study. It is critically needed to perform similar study on female subjects in the future because toxoplasmosis usually induces opposite direction shifts in male and female subjects [26], [59].

The third limitation of the present study concerns the fact that the existence of a significant statistical effect does not imply the existence of the real effect of a particular independent variable, e.g. the toxoplasmosis, on a dependent variable, e.g. the intelligence. The observed statistical effect could be caused by an effect of the intelligence on the risk of *Toxoplasma* infection or even by an effect of some unknown third factor on both intelligence and risk of *Toxoplasma* infection.

It is highly probable that similar or even stronger associations could exist between infection with other pathogens, e.g. chlamydia, yeasts and herpetic viruses, and behavioural and psychological traits. For example, not only the infection with *Toxoplasma* but also with human cytomegalovirus is accompanied by decreased Cloninger's personality factor Novelty seeking [60]. Our subjects were not tested for presence of other infectious agents except *Toxoplasma* and therefore we could not include these potential confounding factors into our models. It must be stressed, however, that the absence of these factors in the models could cause false negative but not false positive results of statistical tests.

## Conclusions

The effect of blood groups on personality and intelligence was the subject of many earlier studies. Despite the widespread believe in the existence of such effects in some cultures, e.g. in Japan, rigorous tests usually provided only negative results. It must be reminded, however, that the ABO blood group system rather than the Rhesus factor system was nearly always examined in these studies, see [61]–[64]. Our results imply that in future behavioural studies the attention should be focused not only on the ABO system but also on RhD phenotype and that important confounding variables, especially *Toxoplasma* infection and smoking [47] should be controlled.
